# The Influence of Grain Size on Microstructure Evolution in CeO_2_ under Xenon Ion Irradiation

**DOI:** 10.3390/nano14181498

**Published:** 2024-09-15

**Authors:** Penghui Lei, Xiaoyu Ji, Jie Qiu, Jiaxuan Si, Tao Peng, Changqing Teng, Lu Wu

**Affiliations:** 1 School of Nuclear Science and Technology, Xi’an Jiaotong University, Xi’an 710049, China; penghuilei@xjtu.edu.cn (P.L.); 2192112868@stu.xjtu.edu.cn (X.J.); 2 The First Sub-Institute, Nuclear Power Institute of China, Chengdu 610041, China; sijiaxuan9610@126.com (J.S.); pengtaoustb@163.com (T.P.); 21626040@zju.edu.cn (C.T.)

**Keywords:** grain size, cerium dioxide, ion irradiation, dislocation loops, gas bubbles

## Abstract

Large-grained UO_2_ is considered a potential accident-tolerant fuel (ATF) due to its superior fission gas retention capabilities. Irradiation experiments for cerium dioxide (CeO_2_), used as a surrogate fuel, is a common approach for evaluating the performance of UO_2_. In this work, spark plasma sintered CeO_2_ pellets with varying grain sizes (145 nm, 353 nm, and 101 μm) and a relative density greater than 93.83% were irradiated with 4 MeV Xe ions at a fluence of 2 × 10^15^ ions/cm^2^ at room temperature, followed by annealing at 600 °C for 3 h. Microstructure, including dislocation loops and bubble morphology of the irradiated samples, has been characterized. The average size of dislocation loops increases with increasing grain size. Large-sized dislocation loops are absent near the grain boundary because the boundary absorbs surrounding defects and prevents the dislocation loops from coalescing and expanding. The distribution of bubbles within the grain is uniform, whereas the large-sized and irregularly shaped xenon bubbles observed in the small grain exhibit pipe diffusion along the grain boundaries. The bubble diameter in the large-grained pellet is the smallest. As the grain size increases, the volumetric swelling of the irradiated pellets decreases while the areal density of Xe bubbles increases. Elemental segregation, which tends to occur at dislocation loops and grain boundaries, has been analyzed. Large-grained CeO_2_ pellet with lower-density grain boundaries exhibits better resistance to volumetric swelling and elemental segregation, suggesting that large-grained UO_2_ pellets could serve as a potential ATF.

## 1. Introduction

Irradiation-induced unconventional structural modification of UO_2_ where fission reactions occur is termed high-burnup structure (HBS) [1,2,3]. These structures are characterized by the presence of nanocrystalline grains and micro-pores, which can lead to significant changes in fuel properties [4]. Changes in fuel properties can have a substantial impact on the economic efficiency and operational safety of nuclear power plants. Large-grained UO_2_ is considered a potential ATF due to its superior fission gas retention capabilities and resistance to HBS formation, as well as its enhanced plasticity and thermal creep properties compared to conventional UO_2_ [5,6]. Grain size influences numerous critical mechanisms that can lead to a decline in fuel performance [7]. Therefore, developing novel nanocrystalline UO_2_ fuels that draw inspiration from the HBS is also a promising pathway [8,9]. Inert gases such as xenon, generated by fission reactions and possessing relatively low solubility in nuclear fuel, are easily trapped in grain boundaries or voids, leading to the formation of fission bubbles [10,11,12]. This process degrades the mechanical and thermal properties of the nuclear fuel, resulting in volume swelling, cracking, and decreased thermal conductivity [3]. Therefore, it is crucial to assess and understand the fission gas retention capabilities of UO_2_ with different grain sizes.

CeO_2_ is frequently used as a non-radioactive surrogate fuel for UO_2_ to study microstructural evolution under irradiation due to their nearly identical lattice characteristics, fluorite structure, and similar physical properties (Table 1) [13,14,15,16]. Compared to traditional sintering techniques, Spark Plasma Sintering (SPS) enables the sintering of powder samples into dense pellets without the need for additives at significantly lower temperatures and shorter duration [17,18,19,20,21], which means the growth of grains in the sintered progress is greatly inhibited by SPS. Fission gas migration in fuel is one of the most critical parameters for the economic efficiency and safety of nuclear fuels. Dose rate and temperature can significantly influence the dislocation loop characteristics in Kr^+^-irradiated CeO_2_ during in-situ experiments [15]. According to Yasuda et al. [22], 210 MeV Xe ion irradiation to CeO_2_ induces high-density electronic excitations, leading to microstructural modifications such as dot-contrast features, dislocation loops, line dislocations, and the formation of subdivided grains. CeO_2_ thin films exposed to 92 MeV Xe^23+^ radiation were found to undergo significant microstructural rearrangements and the formation of secondary phases as a result of the high-energy ion irradiation [23]. Xia et al. [24] demonstrated through molecular dynamics and static simulations that the diffusion activation energy and formation energies of Xe atoms at grain boundaries in UO_2_ are significantly lower than those in the bulk. Numerous studies have been published on the impact of grain boundaries on gas migration properties [25,26,27,28], but there is limited information available on the elemental distribution and segregation in SPS-densified pellets with varying grain sizes.

In this work, SPS fabrication was employed to sinter three types of CeO_2_ pellets with different grain sizes. In addition to phase stability, potential CeO_2_ pellets with varying grain sizes should also possess desirable mechanical properties to enhance performance. The effects of grain size on resistance to volumetric swelling, elemental segregation, and dislocation loop formation have been revealed.

## 2. Material and Methods

### 2.1. Preparation of CeO_2_ with Controlled Microstructure

The dense CeO_2_ ceramics with different grain sizes and microstructures were sintered by using SPS technique. Polycrystalline CeO_2_ pellets with different grain sizes (145 nm, 353 nm, and 101 μm) were fabricated by SPS from CeO_2_ powders (99.95% purity, <50 nm, Shanghai Aladdin Biochemical Technology Co., LTD, Shanghai, China). A total of 1.3 g of CeO_2_ powder was loaded into a die with an inner diameter of 10 mm for each sintering process. Nanocrystalline CeO_2_ pellets, with a grain size of 145 nm, were sintered in a tungsten carbide (WC) die for 5 min at 750 °C under a pressure of 120 MPa. The pellets, with a grain size of 353 nm, were sintered in a tungsten carbide (WC) die for 5 min at 1000 °C under a pressure of 120 MPa. The control temperature in these two sintering routes was monitored by a K-type thermocouple. Large-sized CeO_2_ pellets with a grain size of 101 μm were sintered at 1550 °C for 10 min under 40 MPa. To avoid potential interaction between graphite and CeO_2_ during the sintering process, boron nitride spray was applied to the graphite foil wrapped around the inner wall of the die before loading the CeO_2_ powders. A pyrometer was used to monitor sintering temperature above 1000 °C. For all the sintering, the temperature increase rate was set at 100 °C per minute, and an initial pressure of 10 MPa was applied and then linearly increased to the peak pressure with temperatures. The bulk density of the sintered pellets was measured based on Archimedes principle by using an immersing method with deionized water at room temperature (RT) with an Ohaus PX124ZH density profiler (OHAUS Corporation, Pine Brook, NJ, USA).

### 2.2. Irradiation Experiments

**Figure 1:**
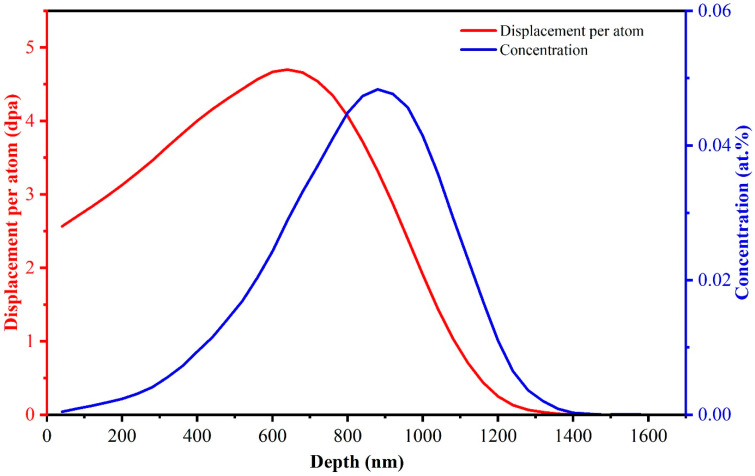
The damage profile and the concentration distribution of implanted Xe ions in CeO_2_ with 4 MeV Xe ions at a fluence of 2 × 10^15^ ions/cm^2^ calculated by SRIM 2013.

The as-sintered CeO_2_ pellets were ground from 400 grid to 5000 grid by using SiC sandpapers, and then polished with 1 μm diamond suspensions. The polished samples were irradiated at room temperature by 4 MeV Xe ions with a fluence of 2 × 10^15^ ions/cm^2^ using the 320 kV platform for multi-discipline research with highly charged ions at the Institute of Modern Physics, CAS (Lanzhou, China). The irradiation damage profile and the depth distribution of implanted Xe ions were calculated using the quick Kinchin–Pease model with the Stopping and Range of Ions in Matter (SRIM) 2013 software [30,31]. The threshold displacement energies for Ce and O elements in cerium dioxide were set to 56 eV and 27 eV, respectively, as reported in reference [32]. The concentration of Xe ions reaches a peak value of approximately 0.05% at a depth of 880 nm, with a displacement damage of 3.35 dpa, as shown in Figure 1. Post-irradiation annealing was performed in a muffle furnace placed in a glovebox. The annealing temperature was set to 600 °C for 3 h to simulate the edge temperature of the pellets during service.

### 2.3. Microstructural Characterizations

XRD was used to study the phase constitution of the sintered pellets. A Gemini SEM 500 SEM (Carl Zeiss AG, Oberkochen, Germany) was used to observe and analyze the fracture surfaces of the samples. Microstructure and morphology characterization using TEM were conducted with a Talos F200X. The cross-sectional TEM samples of the post-irradiation annealed pellet were prepared by focused ion beam (FIB) using a Thermo Scientific Helios 5 UX (Thermo Fisher Scientific, Waltham, MA, USA), achieving a uniform thickness of approximately 100 nm. Micro-indentation testing was carried out to acquire the Vickers microhardness of the sintered samples. Indentation was performed using a diamond-shaped indenter on the polished surface with a load of 1.0 kgf (~9.8N) for 10 s holding, and post indentation measurement was carried out by using SEM. The general approach to estimate the Vickers hardness values is based on the following equation [33]:

$$H=1.8544(\frac{P}{\alpha^{2}})\;[kgf/{mm}^{2}]$$

where *H* is the values of estimated Vickers hardness, α is the average diagonal length of the indentation, and *P* is the applied load.

## 3. Results and Discussion

### 3.1. Microstructure Morphology and Mechanical Properties of the Sintered Samples

**Figure 2:**
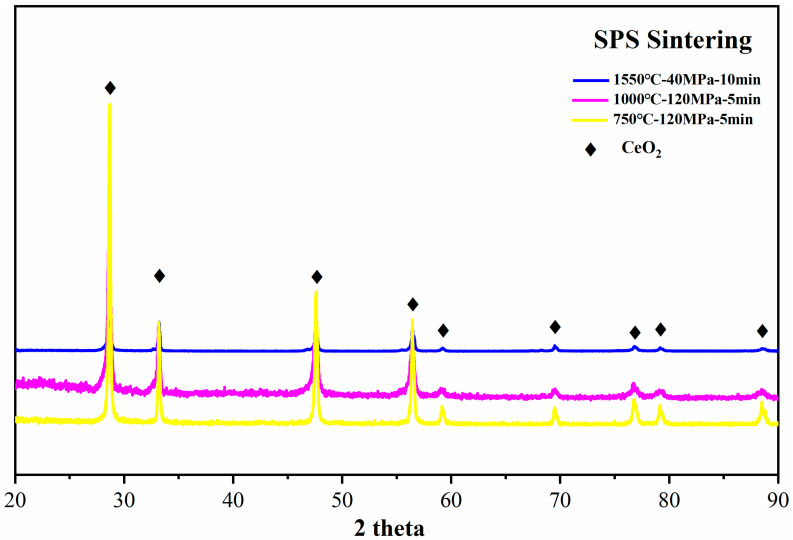
X-ray diffraction patterns of the sintered CeO_2_ pellets from 750 °C to 1550 °C.

The XRD patterns of as-sintered pellets, from 750 °C to 1550 °C, display characteristic diffraction peaks that are well indexed to CeO_2_ as shown in Figure 2. The results indicate that the phase constitution of the sintered pellets by SPS is single-phase CeO_2_, and the stoichiometry of the CeO_2_ sintered pellets remains constant during different SPS sintering temperatures.

**Figure 3:**
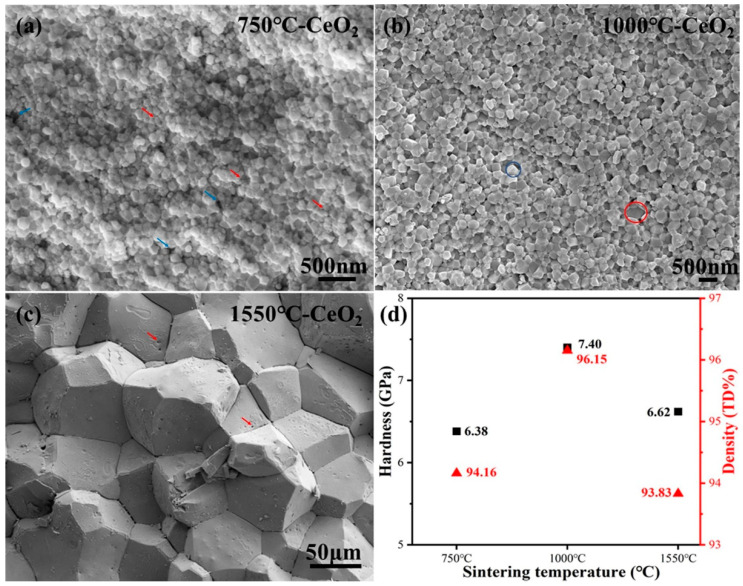
The cross-section fracture surfaces of the sintered CeO_2_ pellets at different sintering temperatures (**a**) 750 °C; (**b**) 1000 °C; (**c**)1550 °C; and Vickers hardness and density of as-sintered CeO_2_ pellets (**d**).

Figure 3a illustrates the cross-sectional fractured surface of the sample sintered at 750 °C for 5 min. It can be observed that the sample contains both small-sized closed pores (indicated by red arrows) and large open pores (indicated by blue arrows), which are distributed around the grain boundaries. The presence of these large open pores inhibits the migration of grain boundaries, thereby decelerating the growth of grains at the same sintering temperature. Moreover, the presence of small-sized closed holes at the triple junction points of grains will serve to reduce the grain coarsening kinetics and restrict grain boundary migration [34,35]. The average grain size of the pellet sintered at 750 °C is 145 nm measured from the SEM image, with a theoretical density of 94.16%. The microstructure of the cross-sectional fractured surface of the sample sintered at 1000 °C for 5 min exhibits similar characteristics, with a much denser theoretical density of 96.15%, as observed in Figure 3b. The average grain size is 353 nm. The occurrence of both trans-granular (indicated by red circle) and intergranular (indicated by blue circle) fracture mechanisms is observed in Figure 3b, and the preferential propagation of cracks is along the weaker grain boundaries [36]. Upon increasing the sintering temperature to 1550 °C for 10 min, significant grain growth is observed, with an average grain size increasing to 101 μm and a theoretical density of 93.83%. Despite the complete elimination of large-sized open pores around grain boundaries, small-sized closed pores (indicated by red arrows) embedded within the grains can still be observed on the fracture surfaces. The fracture surface is characterized by an intergranular fracture model.

The microstructure characteristics of the sintered ceramic samples, particularly the grain sizes and densities, significantly impact their mechanical properties. Since the sintering process involves both the coarsening of the grains and the densification of the material through pore removal, these two variables are interrelated [37]. Grain size and density are found to be highly correlated with the hardness values of CeO_2_ pellets sintered by SPS. As displayed in Figure 3d, the measured hardness initially increases with grain size up to 353 nm and subsequently exhibits a reduction in hardness with further increases in grain size. The pellet sintered at 1000 °C exhibited the highest density (96.15%) and the maximum hardness value (7.40 GPa) among the samples tested. For the pellets sintered at 750 °C and 1550 °C, pores and micro-cracks around the grain boundaries (Figure 3a,c) negatively impact the mechanical integrity. Consequently, much lower hardness values are observed. These results suggest that the elastic and plastic behaviors of as-sintered CeO_2_ pellets upon indentation are mostly determined by grain size and density.

### 3.2. Microstructure of the Irradiated Samples

**Figure 4:**
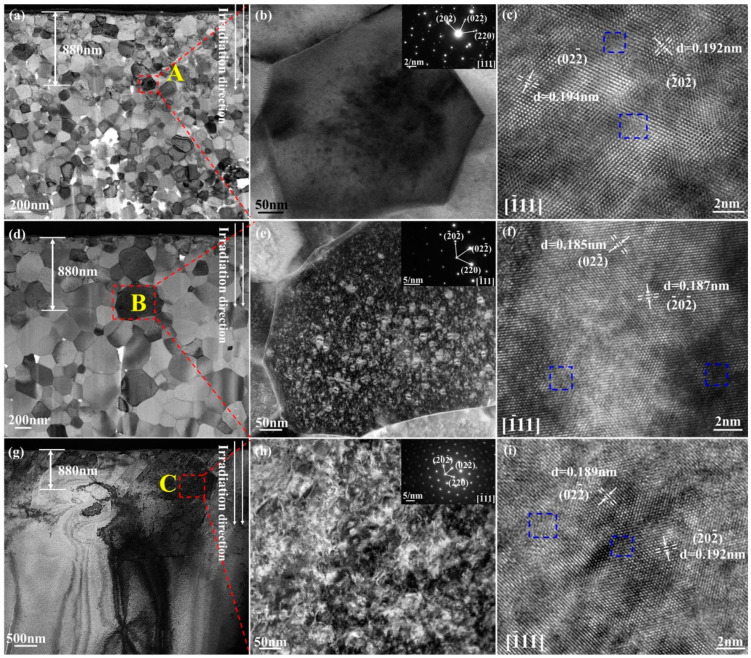
The microstructure of CeO_2_ pellets irradiated with 4 MeV Xe ions with a fluence of 2 × 10^15^ ions/cm^2^ at room temperature followed by annealing at 600 °C for 3 h: (**a**,**c**) the pellet sintered at 750 °C; (**d**–**f**) the pellet sintered at 1000 °C; (**g**–**i**) the pellet sintered at 1550 °C; the SAED patterns of CeO_2_ pellets were inserted in the images (**b**,**e**,**h**).

STEM/TEM images showing the microstructure of CeO_2_ pellets irradiated with 4 MeV Xe ions with a fluence of 2 × 10^15^ ions/cm^2^ at room temperature followed by annealing at 600 °C for 3 h are displayed in Figure 4. exhibits. The incident direction of Xe ions and the peak concentration depth are indicated in the STEM bright field image (Figure 4a) of the pellet sintered at 750 °C. It is evident from the STEM image that these grains in the irradiation area have not undergone amorphization. A grain of approximately 180 nm in size, marked by the red dashed box A in Figure 4a, is subjected to comprehensive analysis in Figure 4b. The grain is situated at a depth of 880 nm below the irradiated surface, with a displacement damage of 3.35 dpa and a peak Xe ion concentration of approximately 0.05%. The selected area electron diffraction (SAED) pattern from the grain is shown in the inserted image in the upper right corner of Figure 4b. The diffraction spots nearest the central spot originate from the (0 2 −2) and (−2 0 −2) crystal planes, with the diffraction zone axis being [–1 1 1], as shown in the insert. The STEM bright field image in Figure 4b, which was taken under the diffraction condition of the [−1 1 1] zone axis, shows the morphology and distribution of dislocation loops in the CeO_2_ after irradiation followed by annealing at 600 °C for 3 h. The average size of dislocation loops is 3.2 nm. Figure 4c presents a high-resolution TEM (HRTEM) image of the peak concentration damage area of the grain for further visualization and structural verification. The atomic-resolution TEM image exhibits a sharp lattice image along the [−1 1 1] zone axis. The measured d-spacings are 0.194 nm and 0.192 nm, which are consistent with the PDF# 34-0394-CeO_2_ (d = 0.191nm) in the Jade database, respectively. Meanwhile, atomic column misalignment regions are observed in the blue dashed box, indicating the presence of dislocation loops and defects caused by irradiation.

The STEM bright field image of the pellet sintered at 1000 °C shows the direction of Xe ions’ incidence and the depth of the peak concentration in Figure 4d. Figure 4e illustrates the results of a grain measuring approximately 600 nm in diameter, as indicated by the red dashed box B in Figure 4d. As indicated in the inset of Figure 4e, the diffraction zone axis of the SAED pattern from the grain is [−1 1 1], and the diffraction spots closest to the center spot originate from the (0 2 −2) and (−2 0 −2) crystal planes. The diffraction condition of the [−1 1 1] zone axis is used to capture the STEM dark field image in Figure 4e, which displays the typical features of dislocation loops with a bimodal size distribution in the CeO_2_ following irradiation and a 3-h annealing process at 600 °C. The dislocation loops have an average diameter of 5.56 nm. The presence of large dislocation loops, with diameters reaching up to 11.80 nm, alongside smaller loops (3.07 nm), suggests that dislocation loop nucleation and growth occurred simultaneously. Large-sized dislocation loops are absent near the grain boundary because the boundary absorbs surrounding defects and prevents the dislocation loops from coalescing and expanding. Grain boundaries have the capacity to function as neutral sinks, whereby they can remove defects through absorption and subsequent recombination of interstitials and vacancies [38]. For additional visualization and structural validation, an HRTEM image of the highest ion concentration region along the [−1 1 1] zone axis of the grain is shown in Figure 4f. The d-spacings of PDF# 34-0394-CeO_2_ (d = 0.191 nm) are in line with the measured d-spacings of 0.185 nm and 0.187 nm, respectively. In the meanwhile, the blue dashed box shows a misalignment of the atomic column.

For the pellets sintered at 1550 °C, microstructure morphologies are shown in Figure 4g. Figure 4h displays the results of a thorough investigation as indicated by the red dashed box C in Figure 4g. The SAED pattern, shown in the inset of Figure 4h, indicates that the diffraction zone axis of the grain is [−1 1 1]. The diffraction condition of the [−1 1 1] zone axis was used to obtain the STEM dark field image shown in Figure 4h, which highlights the characteristic features of dislocation loops in the CeO_2_. The average diameter of the dislocation loops is 10.87 nm. Figure 4i presents an HRTEM image of the region with the highest concentration of Xe ions, oriented along the [1 1 1] zone axis of the grain. The measured d-spacings of 0.189 nm and 0.192 nm are in line with the d-spacings of the PDF# 34-0394-CeO_2_ (d = 0.191 nm), respectively. Meanwhile, atomic column misalignment is indicated by the blue dashed box. It can be concluded that the average size of dislocation loops increases with increasing grain size, consistent with a previous study [39] showing the dislocation loop size in grade 91 steel subjected to Fe ion irradiation at 300 °C also increases with grain size.

### 3.3. Evolution of Gas Bubbles and Element Migration Behavior

**Figure 5:**
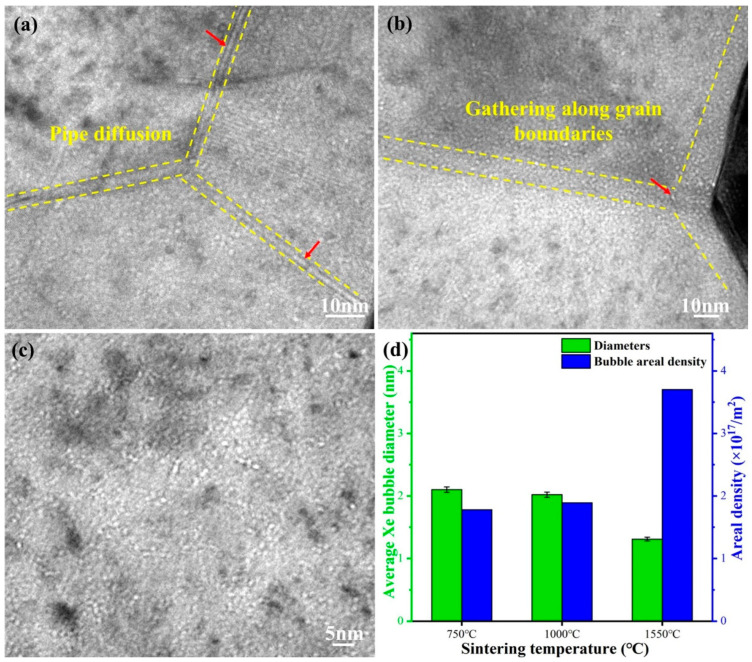
The distribution of Xe gas bubbles in the 4 MeV irradiated CeO_2_ pellets followed by annealing at 600 °C for 3 h: (**a**) the pellet sintered at 750 °C; (**b**) the pellet sintered at 1000 °C; (**c**) the pellet sintered at 1550 °C; and (**d**) average diameters and areal density of Xe bubbles.

The distribution of Xe gas bubbles in the 4 MeV irradiated CeO_2_ pellets, followed by a 3 h annealing at 600 °C, is also investigated with the bubble size and density quantitatively analyzed in Figure 5. The nano-sized bubbles in the sample sintered at 750 °C for 5 min captured using under-focused bright field TEM imaging are illustrated in Figure 5a. It can be observed that the bubbles diffuse along the grain boundaries through pipe diffusion. Xe gas bubbles are uniformly distributed within the grain, with an average diameter of 2.10 ± 0.04 nm. For the pellet sintered at 1000 °C, bubbles gather along the grain boundaries. The distribution of bubbles within the grain is uniform, with an average size of approximately 2.02 ± 0.04 nm. Grain boundaries are prone to trapping bubbles due to their role as defect sinks [25]. The large-sized and irregularly shaped xenon bubbles observed along the grain boundaries in Figure 5a,b are attributed to the pipe diffusion mechanism. This mechanism explains how bubbles grow along grain boundaries, forming coarse and irregular shapes due to the low activation energy and high mobility of the diffusion process [40]. High-density smaller bubbles can be observed through an under-focused TEM image (Figure 5c) showing the morphology and distribution of Xe bubbles. Bubbles are uniformly distributed within the grain, with an average diameter of approximately 1.31 ± 0.03 nm.

Figure 5d presents a histogram describing the bubble diameter and areal densities based on measurements taken across grains of different sizes (Table 2). There is a significant shift in the areal density of Xe bubbles, increasing from an initial 1.78 × 10^17^/m^2^ to a final 3.70 × 10^17^/m^2^, corresponding with increasing grain size. The volumetric swelling rate induced by Xenon bubbles is calculated using the equation provided in reference [41], assuming a thickness of 100 nm for the FIB lamella. The estimated volumetric swelling slightly decreases with increasing grain size, from 0.95% at 145 nm to 0.90% at 353 nm, and then further decreases to 0.49% at 101 μm. The present findings confirm the enhanced resistance to gas precipitation and volumetric swelling in large-grain CeO_2_, in line with the previous report on UO_2_ [26]. The Xe diffusivities at uranium dioxide grain boundaries, as derived from molecular dynamics simulations, exceed those in the bulk by more than six orders of magnitude [27]. Miao et al. highlighted that the excessive dislocations and high density of grain boundaries in UO_2_ may enhance the diffusion of both Xe atoms and vacancies, thereby promoting the formation of micropores [28], which could ultimately result in the cracking of fuel pellets. In large-sized grains, the migration path of fission gas to grain boundaries is longer, making it more difficult for bubbles to diffuse and aggregate. In summary, the formation of small-sized bubbles with a uniform distribution suggests that large-sized CeO_2_ exhibits favorable resistance to volumetric swelling.

**Figure 6:**
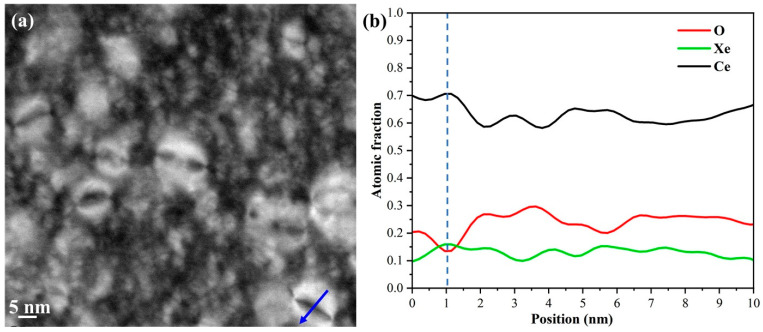
(**a**) A STEM dark filed image showing dislocation loops in irradiated CeO_2_ pellet sintered at 1000 °C; and (**b**) EDS line scan along the blue arrow across the dislocation loop in (**a**).

Irradiation-induced elemental segregation (RIS) at dislocation loops in the 4 MeV irradiated CeO_2_ pellets followed by 600 °C annealing for 3 h was examined using STEM-EDS (energy dispersive spectrometer) analysis. Figure 6 (a) presents a STEM dark-filed image of dislocation loops under the [−1 1 1] zone axis in irradiated CeO_2_ pellets sintered at 1000 °C. The dislocation loops exhibit a bimodal size distribution, with the diameters of the larger loops reaching up to 11.80 nm. To gain more insights into the dislocation loop, EDS line scans were employed to analyze elemental segregation, as shown in Figure 6b. The dislocation loop marked by the blue arrow in Figure 6a indicates that Ce and Xe tend to accumulate around the edge of the loop, while O is depleted in that region. As reported by Cao et al. [14], the dislocation loop in CeO_2_ is considered a strong sink for absorbing point defects and defect clusters induced by irradiation. The observed elemental segregation around the edge of the loop is attributed to the absorption activity of the dislocation loop. Meanwhile, Xe bubbles have accumulated along the grain boundaries, indicating that xenon segregation has also occurred in these regions. Modifications to the chemical composition and atomic structure of grain boundaries can lead to significant changes in a material’s corrosion resistance, radiation tolerance, and imperviousness to fission products [42]. Therefore, large-sized CeO_2_ pellet with lower-density grain boundaries exhibits more favorable resistance to corrosion and elemental segregation.

## 4. Conclusions

In this paper, SPS sintering is applied to fabricate polycrystalline CeO_2_ pellets with different grain sizes (145 nm, 353 nm, and 101 μm) as a surrogate fuel for UO_2_. Radiation-induced microstructure evolution and bubble behavior of CeO_2_ pellets irradiated by 4 MeV Xe ions at room temperature followed by annealing at 600 °C for 3 h are studied. Atomic column misalignment regions are observed in the HRTEM images, indicating the presence of dislocation loops and defects caused by irradiation. The average size of dislocation loops under the [−1 1 1] zone axis increases with increasing grain size of CeO_2_ pellets. The large-sized and irregularly shaped xenon bubbles due to the pipe diffusion mechanism are observed along the grain boundaries in small-sized grains. Bubbles are uniformly distributed within the large-sized grain (101 μm), with an average diameter of approximately 1.31 ± 0.03 nm, the smallest observed in the study. There is a significant shift in the areal density of Xe bubbles, increasing from an initial 1.78 × 10^17^/m^2^ to a final 3.70 × 10^17^/m^2^, corresponding with increasing grain size. The estimated volumetric swelling slightly decreases with increasing grain size, from 0.95% at 145 nm to 0.90% at 353 nm, and then further decreases to 0.49% at 101 μm. Irradiation-induced elemental segregation has occurred around the edge of the dislocation loop and the grain boundary. This work provides a further understanding of how large-sized CeO_2_/UO_2_ pellets exhibit more favorable resistance to volumetric swelling, corrosion, and elemental segregation.

## Figures and Tables

**Table 1:** Summary of the similar properties of UO_2_ and CeO_2_ pellets.

ID	Melting Point (°C)	Fluorite Structure	Thermal Diffusivity	microhardness (GPa)
UO_2_	2870	Yes	Up to 700 °C	6.8–12.9 [29]
CeO_2_	2600	Yes	Up to 700 °C	6.38–7.4 (This work)

**Table 2:** Summary of sintering conditions, average Xe bubble diameters, areal density of Xe bubbles, and estimated swelling of CeO_2_ pellets.

Sintering Temp (°C)	Grain Size	Average Xe Bubble Diameter	Areal Density of Xe Bubble (×10^17^/m^2^)	Estimated Swelling
750	145 nm	2.1	1.78	0.95%
1000	353 nm	2.02	1.89	0.90%
1550	101 μm	1.31	3.7	0.49%

## Data Availability

The data presented in this study are available on request from the corresponding authors.

## References

[1] Matzke H. (1992). On the rim effect in high burnup UO_2_LWR fuels. J. Nucl. Mater..

[2] Xiao H., Long C., Chen H. (2021). The formation mechanisms of high burnup structure in UO_2_ fuel. J. Nucl. Mater..

[3] Jiang Y., La Y., Liu X., Liu W. (2024). Effect of grain size on irradiation-induced bubble evolution in high burn-up UO_2_: A phase-field study. J. Nucl. Mater..

[4] Ronchi C., Sheindlin M., Staicu D., Kinoshita M. (2004). Effect of burn-up on the thermal conductivity of uranium dioxide up to 100.000 MWd t − 1. J. Nucl. Mater..

[5] Kang K.W., Yang J.H., Kim J.H., Rhee Y.W., Kim D.J., Kim K.S., Song K.W. (2010). Effects of MnO-Al_2_O_3_ on the grain growth and high-temperature deformation strain of UO_2_ fuel pellets. J. Nucl. Sci. Technol..

[6] Zhong Y., Gao R., Li B., Yang Z., Huang Q., Wang Z., Duan L., Liu X., Chu M., Zhang P. (2021). Preparation and characterization of large grain UO_2_ for accident tolerant fuel. Front. Mater..

[7] Yu Z., Xu X., Chen W.-Y., Sharma Y., Wang X., Chen A., Ulmer C.J., Motta A.T. (2022). In-situ irradiation-induced studies of grain growth kinetics of nanocrystalline UO_2_. Acta Mater..

[8] Yao T., Mo K., Yun D., Nanda S., Yacout A.M., Lian J. (2017). Grain growth and pore coarsening in dense nano-crystalline UO_2_ + x fuel pellets. J. Am. Ceram. Soc..

[9] Miao Y., Yao T., Lian J., Park J.-S., Almer J., Bhattacharya S., Yacout A.M., Mo K. (2017). In situ synchrotron investigation of grain growth behavior of nano-grained UO_2_. Scr. Mater..

[10] Zacharie I., Lansiart S., Combette P., Trotabas M., Coster M., Groos M. (1998). Thermal treatment of uranium oxide irradiated in pressurized water reactor: Swelling and release of fission gases. J. Nucl. Mater..

[11] Baker C. (1977). The migration of intragranular fission gas bubbles in irradiated uranium dioxide. J. Nucl. Mater..

[12] Fisher S., White R., Cook P., Bremier S., Corcoran R., Stratton R., Walker C.T., Ivison P., Palmer I. (2002). Microstructure of irradiated SBR MOX fuel and its relationship to fission gas release. J. Nucl. Mater..

[13] Ye B., Oaks A., Kirk M., Yun D., Chen W.-Y., Holtzman B., Stubbins J.F. (2013). Irradiation effects in UO_2_ and CeO_2_. J. Nucl. Mater..

[14] Cao Z., He K., Ran G., Qiu X., Sun D., Li Y., Xin Y. (2022). Influence of thermal effect on dislocation loop evolution in Fe^+^-irradiated CeO_2_ during in-situ annealing. Ceram. Int..

[15] Yuan F., Cao Z., Cui D., Xin Y., Li Y., Sun D., Ran G. (2024). Understanding the dose-rate effect on loop characteristics in Kr^+^-irradiated CeO_2_ by in-situ TEM study. Ceram. Int..

[16] Sonoda T., Kinoshita M., Chimi Y., Ishikawa N., Sataka M., Iwase A. (2006). Electronic excitation effects in CeO_2_ under irradiations with high-energy ions of typical fission products. Nucl. Instrum. Methods Phys. Res. Sect. B Beam Interact. Mater. At..

[17] Gong B., Yao T., Lei P., Harp J., Nelson A.T., Lian J. (2020). Spark plasma sintering (SPS) densified U_3_Si_2_ pellets: Microstructure control and enhanced mechanical and oxidation properties. J. Alloys Compd..

[18] Gong B., Yao T., Lei P., Cai L., Metzger K.E., Lahoda E.J., Boylan F.A., Mohamad A., Harp J., Nelson A.T. (2020). U_3_Si_2_ and UO_2_ composites densified by spark plasma sintering for accident-tolerant fuels. J. Nucl. Mater..

[19] Gong B., Cai L., Lei P., Metzger K.E., Lahoda E.J., Boylan F.A., Yang K., Fay J., Harp J., Lian J. (2020). Cr-doped U_3_Si_2_ composite fuels under steam corrosion. Corros. Sci..

[20] Yao T., Gong B., Lei P., Lu C., Xu P., Lahoda E., Lian J. (2020). UO_2_ + 5 vol% ZrB_2_ nano composite nuclear fuels with full boron retention and enhanced oxidation resistance. Ceram. Int..

[21] Guo X., Gin S., Lei P., Yao T., Liu H., Schreiber D.K., Ngo D., Viswanathan G., Li T., Kim S.H. (2020). Self-accelerated corrosion of nuclear waste forms at material interfaces. Nat. Mater..

[22] Yasuda K., Etoh M., Sawada K., Yamamoto T., Yasunaga K., Matsumura S., Ishikawa N. (2013). Defect formation and accumulation in CeO_2_ irradiated with swift heavy ions. Nucl. Instrum. Methods Phys. Res. Sect. B Beam Interact. Mater. At..

[23] Popel A., Le Solliec S., Lampronti G., Day J., Petrov P., Farnan I. (2017). The effect of fission-energy Xe ion irradiation on the structural integrity and dissolution of the CeO_2_ matrix. J. Nucl. Mater..

[24] Xia Y., Wang Z., Wang L., Chen Y., Liu Z., Wang Q., Wu L., Deng H. (2022). Molecular Dynamics Simulations of Xe Behaviors at the Grain Boundary in UO_2_. Metals.

[25] Yan X., Li Z., Lei P., Wang S., Gao R. (2024). Significant suppression of helium bubbles in oxide dispersion strengthened FeCrAl alloys irradiated by high concentration of helium. Vacuum.

[26] Cappia F., Cullison M., Chen T., Kombaiah B., Bawane K., Teng F., Madden J., Perez E., Yao T., Lei P. (2022). Grain subdivision and structural modifications by high-energy heavy ions in UO_2_ with different initial grain size. Nucl. Instrum. Methods Phys. Res. Sect. B Beam Interact. Mater. At..

[27] Liu X.-Y., Galvin C.O., Cooper M.W.D., Andersson A.D.R. (2023). Molecular Dynamics Simulations of Fission Gas Xenon (Xe) Diffusion at UO_2_ Grain-Boundaries (Rev. 1).

[28] Miao Y., Yao T., Lian J., Zhu S., Bhattacharya S., Oaks A., Yacout A.M., Mo K. (2018). Nano-crystallization induced by high-energy heavy ion irradiation in UO_2_. Scr. Mater..

[29] Gong B., Frazer D., Yao T., Hosemann P., Tonks M., Lian J. (2019). Nano-and micro-indentation testing of sintered UO_2_ fuel pellets with controlled microstructure and stoichiometry. J. Nucl. Mater..

[30] Iwamoto Y., Meigo S.-I., Hashimoto S. (2020). Estimation of reliable displacements-per-atom based on athermal-recombination-corrected model in radiation environments at nuclear fission, fusion, and accelerator facilities. J. Nucl. Mater..

[31] Nordlund K., Zinkle S.J., Sand A.E., Granberg F., Averback R.S., Stoller R.E., Suzudo T., Malerba L., Banhart F., Weber W.J. (2018). Primary radiation damage: A review of current understanding and models. J. Nucl. Mater..

[32] Guglielmetti A., Chartier A., van Brutzel L., Crocombette J.-P., Yasuda K., Meis C., Matsumura S. (2008). Atomistic simulation of point defects behavior in ceria. Nucl. Instrum. Methods Phys. Res. Sect. B Beam Interact. Mater. At..

[33] (2019). Standard Test Method for Vickers Indentation Hardness of Advanced Ceramics.

[34] Liao S.C., Mayo W.E., Pae K.D. (1997). Theory of high pressure/low temperature sintering of bulk nanocrystalline TiO_2_. Acta Mater..

[35] Lei P., Yao T., Gong B., Zhu W., Ran G., Lian J. (2020). Spark plasma sintering-densified vanadinite apatite-based chlorine waste forms with high thermal stability and chlorine confinement. J. Nucl. Mater..

[36] Yao T., Scott S., Xin G., Lu F., Lian J. (2015). Dense Iodoapatite Ceramics Consolidated by Low-Temperature Spark Plasma Sintering. J. Am. Ceram. Soc..

[37] Lei P., Yang K., Shi T., Wei M., Ran G., Lu C. (2022). Surface alteration and chemical durability of hollandite (Cr, Al and Ti) consolidated by spark plasma sintering in acid solution. J. Nucl. Mater..

[38] El-Atwani O., Esquivel E., Efe M., Aydogan E., Wang Y., Martinez E., Maloy S.A. (2018). Loop and void damage during heavy ion irradiation on nanocrystalline and coarse grained tungsten: Microstructure, effect of dpa rate, temperature, and grain size. Acta Mater..

[39] Duan J., Wen H., He L., Sridharan K., Hoffman A., Arivu M., He X., Islamgaliev R., Valiev R. (2022). Effect of grain size on the irradiation response of grade 91 steel subjected to Fe ion irradiation at 300 °C. J. Mater. Sci..

[40] Miller M., Kenik E., Russell K., Heatherly L., Hoelzer D., Maziasz P. (2003). Atom probe tomography of nanoscale particles in ODS ferritic alloys. Mater. Sci. Eng. A.

[41] Ukai S., Uwaba T. (2003). Swelling rate versus swelling correlation in 20% cold-worked 316 stainless steels. J. Nucl. Mater..

[42] Wang X., Zhang H., Baba T., Jiang H., Liu C., Guan Y., Elleuch O., Kuech T., Morgan D., Idrobo J.-C. (2020). Radiation-induced segregation in a ceramic. Nat. Mater..

